# Relevance of Cellular Homeostasis-Related Gene Expression Signatures in Distinct Molecular Subtypes of Breast Cancer

**DOI:** 10.3390/biomedicines13051058

**Published:** 2025-04-28

**Authors:** Sharda P. Singh, Chathurika S. Dhanasekara, Michael W. Melkus, Chhanda Bose, Sonia Y. Khan, Flavia Sardela de Miranda, Maria F. Mahecha, Prrishti J. Gukhool, Sahil S. Tonk, Se-Ran Jun, Sahra Uygun, Rakhshanda Layeequr Rahman

**Affiliations:** 1 Department of Internal Medicine and Center of Excellence for Integrated Health, Texas Tech University Health Sciences Center, Lubbock, TX 79430, USA; sahil.tonk@ttuhsc.edu; 2 Department of Surgery and Breast Center of Excellence, Texas Tech University Health Sciences Center, Lubbock, TX 79430, USA; samudani.dhanasekara@ttuhsc.edu (C.S.D.); michael.melkus@ttuhsc.edu (M.W.M.); fsardela@ttuhsc.edu (F.S.d.M.); maria.mahecha@ttuhsc.edu (M.F.M.); prrishti.gukhool@ttuhsc.edu (P.J.G.); 3 Department of Pharmacology and Neuroscience, Texas Tech University Health Sciences Center, Lubbock, TX 79430, USA; chhanda.bose@ttuhsc.edu; 4 Department of Surgery, The University of Texas Rio Grande Valley, Harlingen, TX 78539, USA; sonia.khan@utrgv.edu; 5 Department of Biomedical Informatics, University of Arkansas for Medical Sciences, Little Rock, AR 72205, USA; sjun@uams.edu; 6 Agendia Inc., Irvine, CA 92618, USA; sahra.uygun@agendia.com

**Keywords:** breast cancer, FLEX registry, MammaPrint, BluePrint, gene expression, biomarker

## Abstract

**Background:** Breast cancer is a complex and heterogeneous disease characterized by distinct molecular subtypes with varying prognoses and treatment responses. Multiple factors influence breast cancer outcomes including tumor biology, patient characteristics, and treatment modalities. Demographic factors such as age, race/ethnicity, menopausal status, and body mass index have been correlated with variations in incidence, mortality, and survival rates. Over the past decade, comprehensive genomic profiling has been widely used to identify molecular biomarkers and signatures to develop novel therapeutic strategies for patients. For instance, the FLEX registry (NCT03053193) enrolled stage I–III breast cancer patients across 90 institutions in the United States and stratified risk groups based on a 70-gene signature (MammaPrint^®^-MP) and molecular subtype based on an 80-gene signature (BluePrint^®^-BP). This study aimed to identify the gene expression patterns and biomarkers associated with breast cancer risk and progression by integrating transcriptomic and clinical data. **Methods:** Targeted 111 unique gene expression and clinical data points from 978 breast cancer samples, representing each BP subtype (26% Luminal A, 26% Luminal B, 25% Basal, 23% HER2), obtained from Agendia Inc. These genes were selected based on their involvement in the mercapturic acid pathway, white and brown adipose tissue markers, inflammation markers, tumor-associated genes, apoptosis, autophagy, and ER stress markers. All statistical analyses, including principal component analysis (PCA), were performed using R version [4.4.0]. Prognostic values and genetic alterations were investigated using various web-based programs as described in the Methods section. **Results:** PCA of gene expression data revealed distinct clustering patterns associated with risk categories and molecular subtypes, particularly with principal component 4 (PC4). Genes related to oxidative stress, autophagy, apoptosis, and histone modification showed altered expression across risk categories and molecular subtypes. Key differentially expressed genes included *SOD2*, *KLK5*, *KLK7*, *IL8*, *GSTM1/2*, *GLI1*, *CBS*, and *IGF1*. Pathway analysis highlighted the enrichment of processes related to autophagy, cellular stress response, apoptosis, glutathione metabolism, deacetylation, and oxidative stress in high-risk and basal-like tumors compared with Ultralow and Luminal A tumors, respectively. **Conclusions:** This study identified gene expression signatures associated with breast cancer risk and molecular subtypes. These findings provide insights into the biological processes that may drive breast cancer progression and could inform the development of prognostic biomarkers and personalized therapeutic strategies.

## 1. Background

According to the World Health Organization, breast cancer is the most common cancer affecting women worldwide. In 2024, the American Cancer Association estimated 310,720 new cases of invasive breast cancer, 56,500 new cases of ductal carcinoma in situ (DCIS) and 42,250 deaths from the disease in the United States (US) [1]. Breast cancer is a heterogeneous disease that is biologically characterized by the expression of one or a combination of steroid hormone receptors, such as estrogen receptor (ER), progesterone receptor (PR), oncogene-epidermal growth factor receptor ErbB2 (Her2neu), or absence of expression of ER, PR, and Her2neu receptors (Triple Negative Breast Cancer (TNBC)) [2]. However, this traditional classification system may have limitations for patient-tailored treatment strategies. As a result, gene expression studies have further characterized breast cancer into four molecular subtypes: Luminal A, Luminal B, Her2-enriched, and basal-like/TNBC [3]. TNBC represents 10–15% of all breast cancers and tends to show a more aggressive and worse disease-specific outcome than any other subtype of breast cancer [4]. TNBC can be further classified into four to six subtypes based on the typical gene expression profiles. Still, two basal-like subtypes (mesenchymal and luminal androgen receptor subtypes) have different clinicopathological characteristics and different responses to chemotherapy [5]. Because breast cancer is a highly heterogeneous disease, clinical treatment and prognosis vary greatly between patients. Therefore, identifying the molecular factors that contribute to these differences could lead to more personalized and effective treatment strategies.

Significant disparities exist in breast cancer incidence and mortality among various racial and ethnic groups in the US. For instance, the age-adjusted incidence of breast cancer among non-Hispanic White (NHW) women is 128 per 100,000 [6]. In contrast, the incidence of breast cancer is slightly lower for African-American (AA) and Hispanic-Latino (HL) women (125 and 92 per 100,000, respectively) [4]. Despite the lower incidence of breast cancer, the mortality rate is significantly higher among African Americans and Hispanic Latinos. For instance, the overall five-year survival rate among NHW women is 95%; in contrast, the five-year survival for HL women is 88%, and even worse among AA women (approximately 81%). A precise explanation for such significant racial/ethnic disparities remains unclear; nonetheless, many believe that the problem is multifactorial and might be related to socioeconomic status, limited health care, limited access to health care, and, more likely, different biology of breast cancer among groups. Multiple epidemiological studies have established that TNBC is more prevalent among African-American women than among Hispanic-Latino and non-Hispanic White women [7,8]. However, a population study showed that Hispanic-Latino women, in contrast to non-Hispanic White women, are more likely to initially present with metastatic or advanced breast cancer and, therefore have worse outcomes, suggesting an important role of socioeconomic factors and probably the biology of the tumor [9]. Germani et al. identified 17 deleterious BRCA (BReast CAncer gene) mutations among BRCA 1 and BRCA 2 carriers with breast cancer; notably, they identified five new mutations that were previously unreported in the general population of women with breast cancer [10]. A recent study of tumor samples from 88 Hispanic women with breast cancer from the West Texas region revealed that they present at a younger age and are predominantly TNBC tumors with 20% carrying BRCA mutations [11]. Given that this group only focused on the BRCA genes, it is likely that these patients would have other confounding mutations that have gone undetected. Similarly, our preliminary data suggest that the most common genetic alterations among Hispanic-Latino women with metastatic TNBC are mutations in *TP53* (100%), *NOTCH* (44%), *AKT*, *MAP3K1* (28%), *PIK3CA*, and *EGFR* (20%). In contrast, larger cancer databases such as TCGA (The Cancer Genome Atlas) and Catalogue of Somatic Mutations in Cancer (COSMIC) reported the frequency of *TP53* (83% and 51%), *NOTCH* (N/R and 6.3%), *AKT* (N/R and 2.5%), *MAP3K1* (N/R and 2.5%), *PIK3CA* (8.6% and 13.8%), and *EGFR* (N/R and 1.9%). It is important to note that most patients in larger databases were non-Hispanic White and only a small proportion were African Americans or Hispanic Latinos. Hence, molecular drivers of breast cancer may be different and less explored in Hispanic-Latino and African-American women. Thus, a better understanding of differentially expressed genes and mutations may help to address some of the disparities in outcomes in these groups of patients.

Therapeutic approaches for breast cancer include systemic chemotherapy alone or in combination with immunotherapy administered either before surgery (neoadjuvant chemotherapy, NAC) or following surgery (adjuvant chemotherapy, ACT). NAC offers several advantages, including the potential for breast-conserving surgery, and serves as an in vivo tool to assess tumor sensitivity to chemotherapy. However, the response rate to conventional NAC is approximately 30–40%, with recent studies demonstrating improved response rates with more complex regimens including immunotherapeutic agents. For instance, the recently published results of the KEYNOTE 522 clinical trial showed a significantly higher response, approaching 66% [12]. However, the regimen used in this study was much more complex and toxic than conventional CT. Therefore, the major challenge for medical oncologists is to empirically estimate the risk/benefit balance and determine which patients will benefit most from potentially toxic NAC. Earlier, we did not have an objective standard tool to calculate risk/benefit scores most of the time, and such decisions were based on individual tumor clinicopathological features, grade, size, tumor stage, hormonal receptor status, and medical oncologist experience. It would be crucial for medical oncologists to have a standard objective tool or test to identify tumors sensitive to chemotherapy before treatment initiation. Furthermore, it is important to understand which tumors are partially resistant to chemotherapy and might require a more extended course of chemotherapy or multi-agent chemotherapy combined with immunotherapy.

Currently, several genomic tests analyze genes that stratify breast tumors that better predict treatment efficacy and risk of recurrence. MammaPrint^®^ (MP) is a 70-gene signature that stratifies early-stage breast cancer patients into low- and high-risk groups for distant relapse. Further stratification of MP risk results identifies four risk subgroups: Ultralow, Low, High 1, and High 2, with specific prognostic and predictive outcomes. BluePrint^®^ (BP) is an 80-gene signature that classifies breast tumors into Luminal A, Luminal B, HER2, or Basal molecular subtypes, providing information on tumor behavior, long-term prognosis, and response to systemic therapy.

We hypothesized that integrating molecular profiling using MP and BP genomic signatures with clinical characteristics, such as age, racial background, BMI, and menopausal status, would reveal distinct breast cancer biology across different patient groups. This integrated approach will improve the prediction of chemotherapy responses, allowing for more personalized and effective treatment strategies. Furthermore, dynamic tumor evaluation during systemic therapy may identify early indicators of treatment response or resistance, enabling therapy adjustments that minimize toxicity while maximizing efficacy. Our main objective was to identify the gene expression patterns associated with risk subgroups, molecular subtypes, and patient-level characteristics.

## 2. Methods

### 2.1. Patient Population and Samples

In the present study, we obtained transcriptomic profiles of breast tumors from Agendia Inc., Irvine, CA, USA) through random selection to represent each molecular subtype equally. All participants provided written informed consent at their institutions before enrollment. The FLEX registry (NCT03053193) enrolls patients with stage I–III breast cancer who receive MP (with or without BP) as the standard of care and consent to whole transcriptome and clinical data collection [13]. Approximately 17,000 patients have been enrolled in FLEX as of 11 September 2024. Transcriptome was based on microarray probe intensities, stratified risk groups were based on the MP, and molecular subtypes were based on the 80-gene signature BP [13]. We obtained transcriptomic data from 172 microarray probes corresponding to 111 unique genes involved in the mercapturic acid pathway, white and brown adipose tissue markers, inflammation markers, tumor-associated genes, apoptosis, autophagy, and ER stress markers, and clinical data from 978 patient samples, representing each BP subtype (26% Luminal A, 26% Luminal B, 25% Basal, 23% HER2). Of the 978 patients (White: 81.7%; Black: 12.58%; other races: 5.7%), the risk or genetic profile was as follows: MP: Ultralow = 76 (8%), Low = 176 (18%), High 1 = 315 (32%), and High 2 = 411 (42%); BP: Luminal A = 250 (26%), Luminal B = 250 (26%), HER2 = 228 (23%), and Basal = 250 (26%) (Table 1).

### 2.2. Principal Component Analysis of Gene Expression Data

All statistical analyses were performed using R version [4.4.0] (https://cran.r-project.org/) last accessed on 15 October 2024. Before the analysis, the gene expression data underwent preprocessing steps to ensure quality and consistency. First, to stabilize the variance and normalize the distribution of the expression levels, the data were log-transformed. Next, the log-transformed expression values were standardized to have a mean of 0 and standard deviation of 1 using the scale function in R. Principal component analysis (PCA) was performed to identify the key patterns of variation across samples and visualize them in a lower dimensional space. The PCA was conducted using the ‘PCAtools’ package in R. The preprocessed gene expression matrix, which consisted of 110 genes and 978 samples, was used as the input for the PCA. The median signal of the probes was calculated when multiple probes, which correspond to the same genes, were used. The PCA algorithm decomposes the scaled data matrix into principal components (PCs), capturing the maximum variance in the data. Genes with low variance (<10%) across samples were filtered to focus on the most informative features. A scree plot was generated to visualize the proportion of variance explained by each PC. The elbow method was used to determine the number of PCs that needed to be included in the downstream analysis. The loadings (eigenvectors) and variance explained (eigenvalues) for each PC were extracted to interpret the contribution of each individual gene to the PCs. Genes responsible for explaining the top 1% of the variance captured by each PC were visualized on the PCA biplot. Further, we visualized the correlation between the PCs and clinical variables, using a heatmap. The family-wise error rate was set at 0.05, using the Holm–Bonferroni approach. Analysis of Variance (ANOVA) was performed to test the differences between the groups using the scores of the first five PCs. The variables considered for group comparisons were race, menopausal status, MP risk categories (i.e., Ultralow, Low, High 1, and High 2), and BP molecular subtypes (i.e., Luminal A, Luminal B, HER2, and Basal). For continuous variables, such as age and body mass index (BMI), linear regression models were constructed using the scores of the first five PCs.

### 2.3. Differential Gene Expression Analysis

Differential gene expression analysis was performed using R software [4.4.0] to identify differentially abundant genes. *t*-Test was used to compare each microarray probe’s mean expression between MP risk categories, using Ultralow as a control, and between BP molecular subtypes, using Luminal A as a control. Multiple testing correction was done using Benjamini–Hochberg. For statistical analysis and visualization, we used tidyverse, and the ComplexHeatmap package was implemented in R 4.4.0. Overall survival (OS) analysis of selected differentially expressed genes (DEGs) was performed using the KMplotter web portal (https://kmplot.com/analysis/index.php?p=service) last accessed 15 October 2024.

### 2.4. Gene Ontology (GO) and KEGG Pathway Enrichment Analysis

Pathway enrichment was performed using Metascape (https://metascape.org/) last accessed on 15 October 2024 considering Gene Ontology (GO) Biological Process terms and Hallmark gene sets. Genes used were significantly up- or down-regulated DEGs (with a *p*-value < 0.05) from comparing MP High 2 to Ultralow and BP Basal to Luminal A to identify characteristic biological attributes stratified into higher categories. Kyoto Encyclopedia Gene and Genome (KEGG) analysis of the DEGs (with a *p*-value < 0.05) was performed online with the bioinformatic tool KEGG Mapper. The Database for Annotation, Visualization, and Integrated Discovery (DAVID) tool was used to uncover the genes’ biological processes and molecular functions.

### 2.5. Protein–Protein Interaction (PPI) Network Analysis

The STRING database (http://string-db.org and last accessed on 15 October 2024) is a commonly used tool for analyzing direct and indirect interactions of proteins. Significantly up- or down-regulated DEGs (*p* < 0.05) from MP risk types or BP molecular subtypes were uploaded to STRING Version 12.0 to analyze the known and predicted interactions.

## 3. Results

### 3.1. Study Population

The study included samples from 978 patients with a mean age of 58.84 ± 13.04 and BMI of 30.04 ± 7.34 kg/m^2^. The majority of patients in all groups were White (*n* = 799, 81.7%), 123 (12.58%) were Black, and the rest belonged to other races (*n* = 56, 5.7%). The majority were post-menopausal females (*n* = 717, 72.94%), while the rest were either pre-menopausal (*n* = 133, 13.53%) or peri-menopausal (i.e., within 6–12 months of the last menstrual period, *n* = 128, 13.02%). The risk or genetic profile was as follows: MP: Ultralow = 76 (8%), Low = 176 (18%), High 1 = 315 (32%), and High 2 = 411 (42%); BP: Luminal A 250 (26%), Luminal B = 250 (26%), HER2 = 228 (23%), and Basal = 250 (26%). Table 1 represents the baseline clinical and demographic data. MammaPrint and BluePrint category distribution of the FLEX samples used in this study is as follows: Luminal A category contains 30.40% Ultralow and 69.60% Low; Luminal B contains 87.20% High 1 and 12.80% High 2; HER2 contains 36.40% High 1 and 62.70% High 2; and Basal category consists of 5.60% High 1 and 94.40% High 2 patients’ samples (Table S1).

### 3.2. Principal Component Analysis

**Figure 1:**
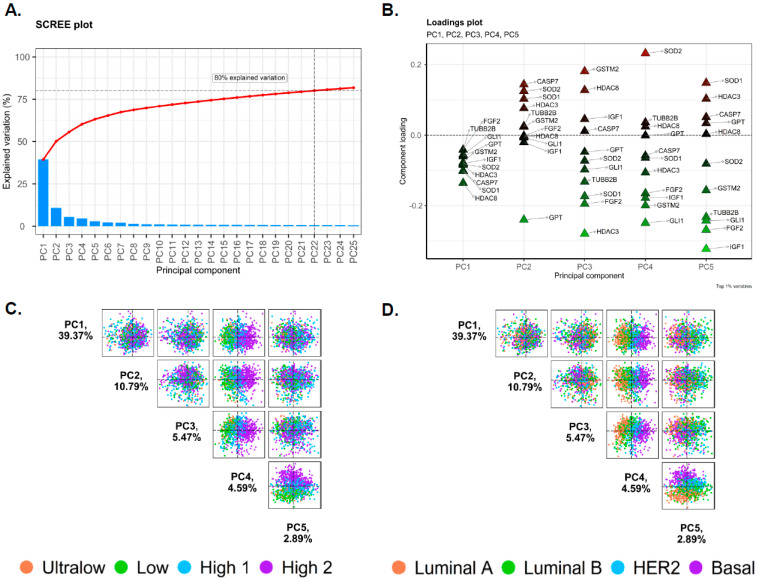
Principal component analysis (PCA). (**A**) Scree plot; the percentage of variation for each principal component (PC) is shown. (**B**) Loadings plot; genes with significant relationships to PC1–5 are shown. (**C**) The pairs plot of risk subtypes; the pairs plots show the percentage of variance explained by each PC for the top five PCs. The colors are based on the risk of recurrence (Ultralow, Low, High 1 and High 2). (**D**) Pairs plot of molecular subtypes; the pairs plot shows the percentage of variance explained by each PC for the top five PCs. The colors are based on distinct molecular subtypes within the dataset (Luminal A, Luminal B, Basal, and HER2 subtypes).

The first two PCs (PC1 and PC2) accounted for a substantial proportion of the total variance, with PC1 explaining 39.37% and PC2 explaining 10.79%, which together accounted for 50.16% of the total variance in gene expression data. The scree plot (Figure 1A) shows the percentage of the variance each PC explains. According to the Elbow method, five PCs were sufficient to retain for the analysis. Together, the first 22 PCs explained approximately 80% of the total variance in the dataset.

The loadings plot displays the contribution of each gene to the PCs, highlighting the key genes (*CASP7*, *FGF2*, *GLI1*, *GPT*, *GSTM2*, *HDAC3*, *HDAC8*, *IGF1*, *SOD1*, *SOD2*, and *TUBB2B*) that drive the observed variance (Figure 1B). For example, the loading plot showed a notable positive loading for *SOD2* in PC4, indicating a significant role of *SOD2* in PC4. These five PC loadings (variable contributes) were enriched for genes associated with various cellular mechanisms, including glucose metabolism, cellular growth, resistance to cell death, angiogenesis induction, oxidative stress handling, and epigenetic modification.

**Figure 2:**
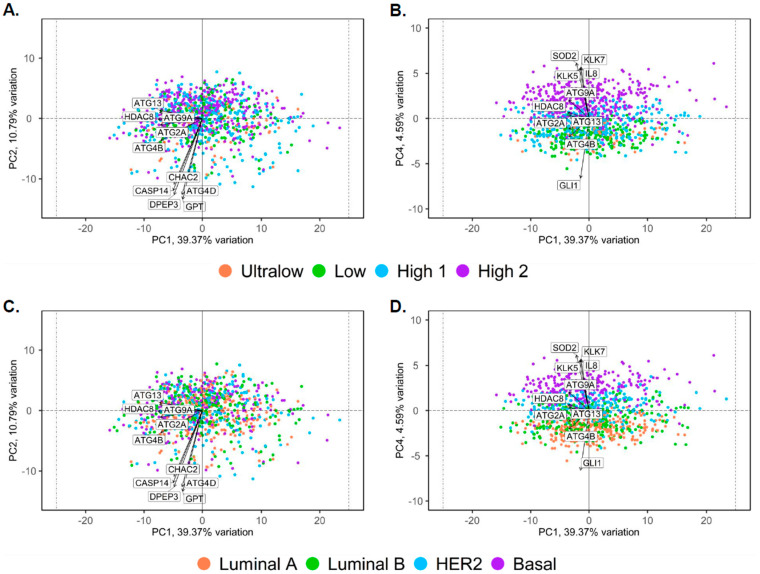
Biplot analysis for gene variance. (**A**) Biplot for PC1 and PC2 denotes gene variance for risk of recurrence (Ultralow, Low, High 1, and High 2 subtypes). (**B**) Biplot for PC1 and PC4 denotes gene variance for risk of recurrence (Ultralow, Low, High 1, and High 2 subtypes). (**C**) Biplot for PC1 and PC2 denotes gene variance for distinct molecular subtypes within the dataset (Luminal A, Luminal B, Basal, and HER2 subtypes). (**D**) Biplot for PC1 and PC4 denotes gene variance for distinct molecular subtypes within the dataset (Luminal A, Luminal B, Basal, and HER2 subtypes). The biplot illustrates the distribution of samples based on principal component 1 (PC1) and PC2, which together accounted for 50.16% of the total variance in the dataset, along with PC1 and PC4 comparison, which showed a certain degree of clustering. The axes represent these PCs, with PC1 on the X-axis and PC2 or PC4 on the Y-axis. Each point in the plot corresponds to an individual sample. Each arrow indicates a gene. The direction and length of the arrows indicate the contribution of each gene to PC. Longer arrows indicate stronger contributions to the PCs. Genes pointing in the same direction were positively correlated, whereas those pointing in opposite directions were negatively correlated.

Interestingly, the pairs plot comparing PC1 through PC5 showed a certain degree of clustering, and PC4 (4.59% of the total variance) indicated distinct clustering for each molecular subtype and risk category (Figure 1C,D). The biplot of PC1 and PC2 illustrated the distribution of samples and several genes which contributed to the variance captured by PC1 and PC2, where the breast cancer samples were not clustered into distinct groups, indicating minimal heterogeneity within the dataset when considering PC1 and PC2 (Figure 2A,C). However, several genes were annotated in the plot, indicating their significant contributions to the variance captured by PC1 and PC2. For instance, genes such as *ATG13*, *HDAC8*, *ATG9A*, *ATG4B*, *CHAC2*, and *GPT* were positioned away from their origin, signifying their substantial influence on these PCs. The direction and distance from the origin reflect the magnitude and nature of their contribution. For example, *ATG13* and *HDAC8* had strong negative loadings on PC1, whereas genes such as *CHAC2*, *CASP14*, and *GPT* showed notable influences on PC2. The biplot for PC1 and PC4 showed a certain degree of segregation when the intrinsic molecular subtypes and risk of recurrence categories were considered (Figure 2B,D). *SOD2*, *KLK5*, *KLK7*, and *IL8* showed a strong positive correlation with PC4, whereas *GLI1* showed a strong negative correlation with PC4.

**Figure 3:**
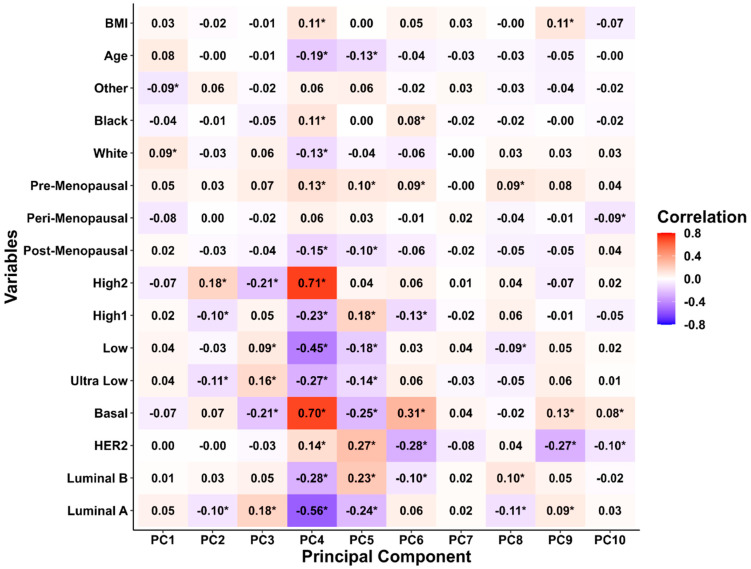
Correlation heatmap between principal components (PC1PC10) and variables. Correlations between PC1–10 and age, body mass index (BMI), race, menopausal status, risk of recurrence, and intrinsic molecular subtypes. The color of each tile represents the strength and direction of the correlation, with red indicating a positive correlation, blue indicating a negative correlation, and white indicating no correlation. The color gradient ranged from blue (−0.8), to white (0), to red (0.8). The numerical values within the tiles represent the correlation coefficients between the variables and PCs. Correlation values annotated with an asterisk (*) indicate statistical significance after adjusting for multiple comparisons using the Holm–Bonferroni approach. The significance level was set at *p* < 0.05.

**Figure 4:**
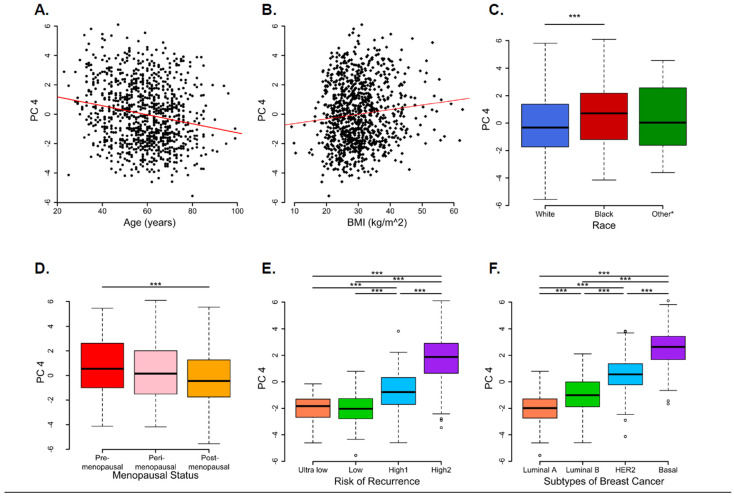
Relationship between principal component 4 scores and baseline variables. Variables considered for group comparisons were (**A**). age, (**B**). body mass index (BMI), (**C**). race (other* includes Asian, Native American, Hispanic, Hawaiian, or Pacific Islander), (**D**). menopausal status, (**E**). MammaPrint, the 70-gene signature, categories of risk of recurrence, and (**F**). BluePrint-80-gene expression-based intrinsic molecular subtypes. Box plots annotated with an asterisk indicate statistical significance after adjusting for multiple comparisons using the Holm–Bonferroni approach. The significance level was set at (asterisk) *p* = 0.05, *** *p* < 0.05.

Next, we compared PCs with the clinical variables (Figure 3). The heat map shows that PC4 was significantly correlated with various baseline clinical characteristics. Specifically, according to the ANOVA and linear regression analyses, menopausal status (*F*_2,975_ = 3.723, *p* = 0.025), race (*F*_2,975_ = 5.03, *p* = 0.007), and age (*β* = 0.038, *p* = 0.012) were significantly associated with PC1 (Figure S1A, Tables S2 and S3). Intrinsic molecular subtypes and risk categories had a significant effect on PC2. Molecular subtypes (*F*_3,974_ = 3.582, *p* = 0.014) and risk of recurrence (*F*_3,974_ = 13.11, *p* < 0.001) were significantly associated with PC2 (Figure S1B, Tables S2 and S3). Notably, PC4 showed a significant difference in all the variables, including age (*β* = −0.0304, *p* < 0.001), BMI (*β* = 0.031, *p* = 0.001), race (*F*_2,975_ = 8.369, *p* < 0.001), menopausal status (*F*_2,975_ = 12.05, *p* = 0.001), molecular subtype (*F*_3,974_ = 640.7, *p* < 0.001), and risk of recurrence (*F*_3,974_ = 412.7, *p* < 0.001) (Figure 4, Tables S2 and S3).

### 3.3. Differentially Expressed Genes in MP Risk Categories and BP Molecular Subtypes

**Figure 5:**
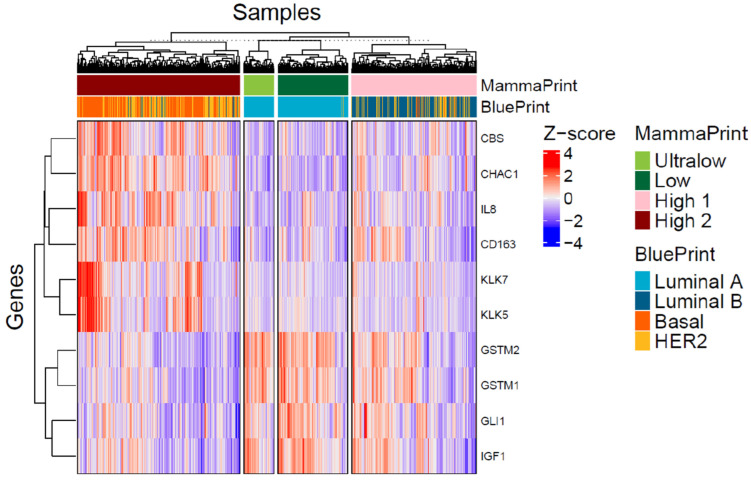
Heatmap of selected genes. Heatmap showing the Z-score of expression of selected genes across FLEX samples. Genes are selected based on top and bottom highest fold change difference between MammaPrint High 2 and Ultralow as well as BluePrint Basal and Luminal A. Columns are genes and rows are samples. Heatmap annotation includes MammaPrint and BluePrint. Clustering of the samples is based on MammaPrint and within each category default hierarchical clustering was used. For the genes, default hierarchical clustering was used within the ComplexHeatmap package.

**Figure 6:**
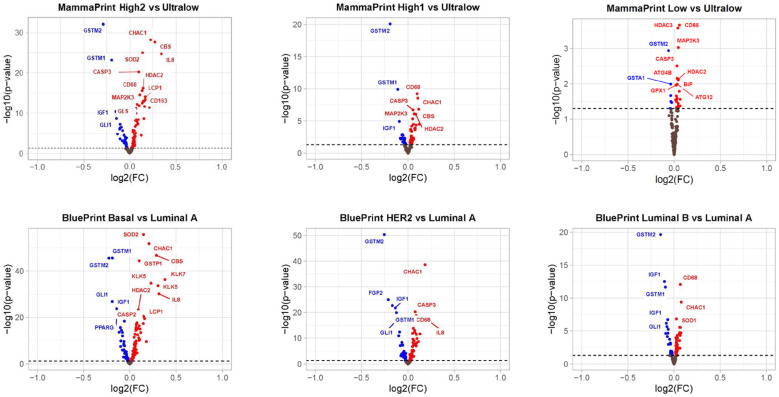
Volcano plots of gene expression comparisons between MammaPrint and BluePrint categories. Compared groups are given in the title of each subplot. The Y-axis is −log10 of the *t*-test *p*-value comparing the mean expression of each probe between MammaPrint and BluePrint categories. The X-axis is the log2 fold change between the mean expressions of probes. Each dot is a gene probe. Dash line is the default cut-off for P value. Top plots, MammaPrint data compared groups High 2 vs. Ultralow, High 1 vs. Ultralow, Low vs. Ultralow. Bottom plots, BluePrint data compared groups Basal vs. Luminal A, HER2 vs. Luminal A, Luminal B vs. Luminal A. Red dots are genes that have a *p*-value < 0.05 and a fold change > 0 (higher expression in High 2/High 1/Low compared to Ultralow or Basal/HER2/Luminal B compared to Luminal A). Blue dots are genes that have a *p*-value < 0.05 and a fold change < 0 (lower expression in High 2/High 1/Low compared to Ultralow or Basal/HER2/Luminal B compared to Luminal A).

**Figure 7:**
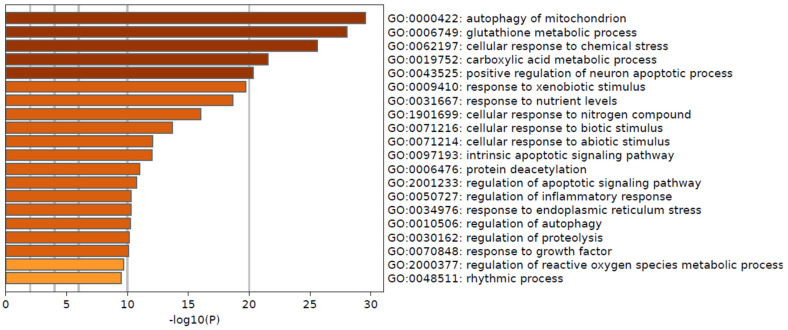
Pathway enrichment for selected genes. Barplot of the top 20 pathway enrichment results. Barplot includes significantly enriched (adjusted *p*-value < 0.05) GO Biological Processes among the 111 input genes using Metascape, https://metascape.org/. The X-axis is the −log10 of the enrichment *p*-value. Color coding correlates to the *p*-value.

**Figure 8:**
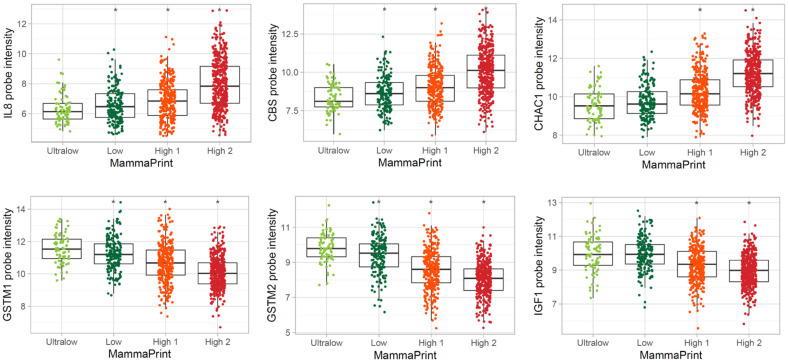
Boxplots of top and bottom three gene expression differences for MammaPrint High 2 and Ultralow category comparison. Genes that have the top three high and low fold changes between MammaPrint High 2 and MammaPrint Ultralow risk categories are in the boxplots. The Y-axis is the log2 probe intensity, X-axis is the MammaPrint categories. * *p* < 0.05, *IL8* (*CXCL8*), *CBS*, and *CHAC1* have highest expression in MammaPrint High 2, while *GSTM2*, *GSTM1*, and *IGF1* have highest expression in MammaPrint Ultralow. Note: inverse correlation of gene expression between High 2 vs. Ultralow.

**Figure 9:**
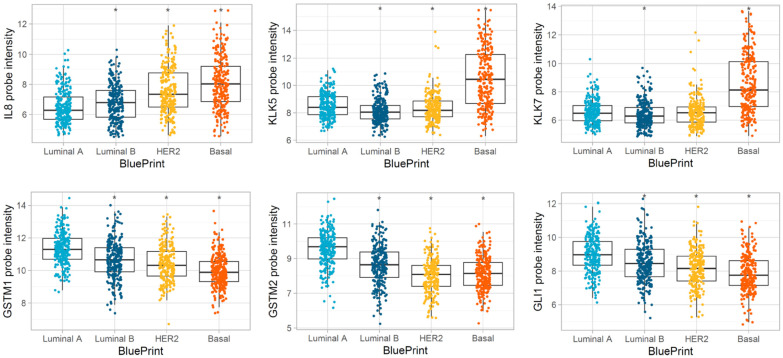
Boxplots of top and bottom three gene expression differences for BluePrint Basal and BluePrint Luminal A category comparison. Genes that have the top three high and low fold changes between BluePrint Basal and BluePrint Luminal A categories are given in the boxplots. The Y-axis is the log2 probe intensity, and the X-axis is the BluePrint categories. * *p* < 0.05, *KLK7*, *IL8* (*CXCL8*), and *KLK5* have the highest expression in BluePrint Basal, while *GSTM1*, *GLI1*, and *GSTM2* have the highest expression in Luminal A. Note: inverse correlation of gene expression between Basal vs. Luminal A.

Gene expression patterns of 111 DEGs were compared between MP risk categories and between BP molecular subtypes. For the MP, we compared: Low vs. Ultralow; High 1 vs. Ultralow; and High 2 vs. Ultralow, considering the group of Ultralow as the control group, and for the BP, we compared: Luminal B vs. Luminal A; Her2 vs. Luminal A; Basal vs. Luminal A, treating the group Luminal A as the control group. For differential abundance analysis, we calculated *p*-values by parametric *t*-tests and log2 fold changes with these comparisons (Table S4). The results were further evaluated/visualized by the heatmap (Figure 5), Volcano plot (Figure 6), Barplot (Figure 7), and Boxplot (Figure 8 and Figure 9).

Unsupervised hierarchical clustering was conducted to search for clusters in significantly altered gene expressions across the samples. Our results show that the tumor samples could be confidently separated into two main groups primarily associated with ER status and risk subgroup (Heatmap Figure 5). *GSTM1*, *GSTM2*, *IGF1*, and *GLI1* clustered together and expressed lower levels in High 2. On the other hand, *KLK5*, *KLK7*, *IL8*, *CD163*, *CBS*, and *CHAC1* are overexpressed depending on MP subtypes.

A comparison was made using parametric *t*-tests indicating that out of 111 genes with a *p*-value < 0.05, 67 DEGs were over-expressed and 28 were under-expressed in BP, and 51 DEGs were over-expressed and 24 were under-expressed in MP (Table S4). When we filtered data by fold change > 0 and plotted against −Log_10_(*p*-value) (Volcano plot Figure 6, the number of DEGs was increased with the complexity of the tumor. The most downregulated genes with a *p*-value < 0.05 and a log2 fold change lower than −1 were *PPARG*, *IGF1*, *GLI1*, *GSTM2*, and *GSTM1* in Basal (BP-subtype), and *IGF1*, *GLI1*, *GSTM2*, and *GSTM1* in High 2 (MP-subtype). On the other hand, higher expression genes with a *p*-value < 0.05 were *SOD2*, *CHAC1*, *CBS*, *GSP1*, *KLK5*, *KLK7*, *HDAC2*, *CASP2*, and *LCP1* in Basal (BP-subtype), and *CHAC1*, *CBS*, *SOD2*, *IL8*, *CASP3*, *HDAC2*, *CD68*, *LCP1*, *MAP2K3*, *CD163*, and *GLS1* in High 2 (MP-subtype).

The three genes with the largest positive fold changes between MP High 2 and MP Ultralow categories were *IL8* (*CXCL8*), *CBS*, and *CHAC1*. Those three genes showed a sequential increase in intensity according to the order of recurrence risk groups (top three plots in Figure 8). *GSTM2*, *GSTM1*, and *IGF1* were the three genes with the largest negative fold changes between MP High 2 and MP Ultralow categories and showed a sequential decrease in intensity according to the order of recurrence risk groups (bottom three plots in Figure 8).

In the comparison of BP Basal and Luminal A categories, *KLK7*, *IL8* (*CXCL8*), and *KLK5* have the largest positive fold changes (top three plots in Figure 9), and GSTM1, *GLI1*, and *GSTM2* have the largest negative fold changes (bottom three plots in Figure 9). Those genes showed a sequential increase or decrease in intensity according to the order of molecular subtypes, except for *KLK5* and *KLK7*. These two genes showed similar average intensity among Luminal A, Luminal B, and HER2 subtypes and a significant increase in Basal.

Seven of nine DEGs were correlated with overall survival, of which the under-expression of *GSTM2*, *GSTM1*, *GLI1*, and *IGF1*, and the overexpression of *IL8* (*CXCL8*), *CBS*, and *CHAC1* were correlated with poor overall survival. *KLK7* and *KLK5* were under-expressed in Luminal B and Her2 but over-expressed in the Basal group, which do not match with overall survivals (Supplemental Figures S2 and S3).

### 3.4. Pathways Differentially Modulated in MP and BP Subgroups

To highlight the physiological processes that could be related to the worsening of breast cancer, we performed KEGG pathway and GO enrichment analysis of DEGs with a *p*-value < 0.05. The GO enrichment analysis showed that biological processes modulated in Basal or High 2 patients belonging to autophagy, cellular response to chemical stress, apoptosis, and glutathione metabolism were significantly enriched (BH adjusted *p*-value < 0.05) (Figure 7 and Table 2). With significantly differentially abundant genes in the comparison of MP High 2 and Ultralow and the comparison of BP Basal and Luminal A, the KEGG pathway analysis revealed that pathways enriched were related to glutathione metabolism, carcinogenesis, cancer pathways, drug metabolism, and metabolic pathways (Tables S5 and S6).

### 3.5. Protein–Protein Interaction Network Associated with Functional Enrichment Analysis of DEGs

DEGs with a *p*-value lower than 0.05 were further analyzed by the STRING database of known and predicted protein–protein interactions to determine the possible interactions among their protein products. Protein products of 67 over-expressed and 28 under-expressed genes in the comparison of BP Basal and Luminal A, and 51 over-expressed and 24 under-expressed genes in the comparison of MP High 2 and Ultralow, were incorporated and used to build the interaction matrix. Eighteen edges (protein–protein interaction) have a high confidence score with six clusters identified for upregulated DEGs in those comparisons (Table S4). The proteins of the largest cluster belong to the categories of autophagy, cellular response to stress, apoptotic process, glutathione metabolism, histone deacetylase, and ROS metabolic process. Furthermore, there are proteins that, although they do not interact with each other, belong to the Cyclooxygenase 2 (*COX-2*) associated genes (*KLK5*, *KLK7*), membrane-bound dipeptidase (DPEP2) and tubulin structure development (Supplemental Figures S6 and S7). When we analyzed downregulated DEGs in those comparisons, we found that they formed three major clusters belonging to the positive regulation of muscle function, autophagy, and drug metabolism.

## 4. Discussion

The genetic characteristics that dictate lineage infidelity during carcinogenesis are not adequately understood. The onset of different stresses, including metabolic and oxidative stress, accompanies oncogene-induced hyper-proliferation in cancer cells. For instance, excessive accumulation of reactive oxygen species (ROS) dictates a multitude of cell-signaling pathways to facilitate the malignant transformation of tumor cells. Oxidative burden in tumor cells mandates reinforcing antioxidant capacity to mitigate detrimental damages. However, their anti-apoptotic effects could negate this benefit, allowing genetically damaged cells to survive and become malignant. However, most reported studies involve gene expression comparisons between normal/non-cancerous versus cancerous tissues or untreated patient tissues versus treated patient tissues [14,15]. Furthermore, the involvement of oxidative stress, histone modification, inflammation, apoptosis, and autophagy in all stages of tumorigenesis, including initiation, promotion, and progression, is still a poorly understood area. Therefore, for the first time using molecular and bioinformatic approaches, we examined whether alterations in gene expression of these pathways can influence the risk of recurrence (Ultralow, Low, High 1, and High 2), and progression of molecular subtypes (Luminal A, Luminal B, HER2, and Basal) in breast cancer [13]. These risk categories and molecular subtypes are derived from transcriptomic analysis of 70 genes (MammaPrint), and 80 genes (BluePrint) that were selected with artificial intelligence-based algorithms by Agendia Inc. based on distant metastatic potential at 5 years in untreated patients. This study dives further into differentially regulated genes within these subtypes to identify potential targetable pathways and biomarker value beyond prognostication, which was the sole criteria for original gene selection. Therefore, despite the limitation of identifying stress-related pathways among tumors, that have already been selected based on aggression, this analysis may still be clinically valuable. Tumors in each subcategory vary in their response to chemotherapy, thus, we used the Ultralow group as a control group (to compare Low, High 1, and High 2) and Luminal A as a control group (to compare Luminal B, HER2, and Basal) for gene expression analysis. By integrating clinical characteristics, we further correlated these molecular features to risk subgroups, molecular subtypes, age, BMI, race, and menopausal status. A total of 111 genes were selected, which were related to autophagy (17 genes), histone deacetylase complex (10 genes), glutathione metabolic process (14 genes), cancer-related (20 genes), and apoptosis (12 genes); the rest of the genes belong to inflammation regulation, oxidative stress, and fatty acid metabolism; a few others belong to key cancer-related pathways. Pathological, computational, and bioinformatics techniques for assessing the risk and progression of breast cancer are standard techniques; therefore, we used PCA, DAVID, STRING, GO analysis, and KEGG analysis tools to analyze the transcriptomic data. We found that gene expression levels were altered related to autophagy, apoptosis, histone deacetylation, and oxidative stress, and these were mainly increased with the worsening of the cancer.

PCA is a classic dimension-reduction tool used to analyze differentially expressed genes and their correlation with other clinical characteristics [16,17]. In this study, we examined possible correlations between PCs and contributions from age, BMI, race, menopausal status, intrinsic molecular subtypes, and risk of recurrence. Loading plot (Figure 1B) from PCA analysis showed that the genes with the highest positive or negative loadings for the top 5 PCs (PC1–5) were as follows: *CASP7*, *FGF2*, *GLI1*, *GPT*, *GSTM2*, *HDAC3*, *HDAC8*, *IGF1*, *SOD1*, *SOD2*, and *TUBB2B*. Comprehensive functional annotation clustering using DAVID (Database for Annotation, Visualization, and Integrated Discovery) suggests that these genes are clustered for oxidative stress (*GSTM2*, *SOD1*, *SOD2*), carcinogenesis (*GLI1*, *CASP7*, *FGF2*, *GSTM2*, *IGF*), and transcription regulation (*GLI1*, *HDAC3*, *HDAC8*). *HDAC8* in PC1; *GPT* in PC2; *HDAC3*, *FGF2*, and *SOD1* in PC3; *GLI1*, *GSTM2*, and *IGF1* in PC4; and *IGF1*, *FGF2*, *GLI1*, and *TUBB2B* showed strong negative correlations. On the other hand, *CASPAS7* and *SOD2* in PC2; *GSTM2* and *HDAC8* in PC3; *SOD2* in PC4; and *SOD1* in PC5 showed a strong positive correlation with various cellular functions. The pairs plot comparison of different combinations of PCs by plotting them against each other showed PC1, PC2, PC3, and PC5 had minimal visual clustering, but PC4 (total variance of 4.59%) indicated distinct clustering (Figure 1C,D). We can identify exclusive patterns, clusters, and relationships between different dimensions of the reduced data for High 2 and Basal, allowing us to identify potential separations or differences between groups within the data set.

In biplot illustrations, the direction and distance of arrows from the origin reflect the magnitude and nature of their contribution. The biplot distribution of PC1 and PC2 suggests that breast cancer samples were not clustered into distinct groups, indicating minimal heterogeneity within the MP or BP dataset. However, several genes were annotated in the plot: *ATG2A*, *ATG4B*, *ATG4D*, *ATG9A*, *ATG13*, *HDAC8*, *CASP14*, *DPEP3*, *CHAC2*, and *GPT* were positioned away from their origin, signifying their substantial influence on these PCs. *ATG13* and *HDAC8* had strong negative loadings on PC1, whereas genes such as *ATG4B*, *CHAC2*, *CASP14*, *DPEP3*, and *GPT* showed notable influences on PC2. In PC1 vs. PC4 biplot comparison for the significant relationship of MP and BP genes, the plot showed that *SOD2*, *KLK5*, *KLK7*, and *IL8* have a strong positive influence, whereas *GLI1* and *ATG4B* show a strong negative impact on PC4, and *ATG9A*, *HDAC8*, *ATG13*, and *ATG2B* have moderated influence on PC4. *KLK5*, *KLK7*, and *IL8* are Cyclooxygenase 2-(*COX-2*) associated genes whose expression correlates with aggressive TNBC features and resistance to COX-2 inhibitors [18,19]. KLK5 is considered the physiological activator of KLK7 and both serve as serological biomarkers, indicators of poor prognosis, and have a role in tumor invasion and angiogenesis in breast cancer [20,21]. IL-8 in breast cancer cells stimulates osteoclasts to resorb bone, which triggers bone metastases [19]. Cancer cells are known to have elevated ROS regulated by the SODs, and SOD2 plays a role in activating different signaling pathways, regulating the angiogenic abilities of breast tumor cells [22]. Furthermore, ATG4B, ATG9A, ATG13, and ATG2B levels varied across breast cancer subtypes, but did not show prognostic significance. The role of HDAC8 in cancer remains largely undefined; studies suggest that the expression of *HDAC8* was upregulated in breast cancer tissues as compared to the controls. Glioma-associated oncogene 1 (GLI1) is a critical transcriptional factor of the sonic hedgehog pathway that tends to have higher expression in progressive stages and is related to the unfavorable prognosis of breast cancer [23]. Comparative high expression of *KLK5*, *KLK7*, and *IL8* genes in the Basal and High 2 subtypes correlate with their bad prognosis.

In our correlations analysis of PCs1–10 with age, BMI, race, menopausal status, intrinsic molecular subtypes, and risk of recurrence, only PC4 showed significantly different associations with all the variables. Here, High 2 and Basal type cancer indicated a significant positive correlation, while Ultralow and Luminal A type breast cancer indicated a significant negative correlation. Interestingly, menopausal status, race, and age were significantly associated with PC1, and the molecular subtypes and risk of recurrence were significantly associated with PC2, PC3, PC5, and, most significantly, PC4. Our PCA analysis suggests that *SOD2*, *KLK5*, *KLK7*, and *IL8* play a significant role in the worsening of breast cancer pathology. PC4 showed a significant differential effect among all PCs despite contributing only 4.59% variability, and bears strong associations with the clinical outcome. This suggests that PCA contributed to delineating hidden associations by reducing the dimensionality of data.

GO enrichment analysis suggested that most DEGs belonged to autophagy, cellular response to chemical stress, apoptotic process, glutathione metabolic process, deacetylation, and ROS. KEGG enrichment analysis (KEGG pathway database), using significantly differentially abundant genes identified after comparing High 2 vs. Ultralow or Basal vs. Luminal A, that autophagy, glutathione metabolism, carcinogenesis, drug metabolism, and cancer pathways are the major pathways. Functional enrichment analysis of significantly differentially upregulated genes using PPI network analysis showed five to six principal main clusters related to histone deacetylation, apoptotic signaling, drug metabolism, glutathione metabolism, autophagy, and drug metabolism. Only KLK5, KLK7, and DPEP2 were not functionally associated with any network. This indicates that increased expression of these genes has a cumulative effect on breast cancer progression.

Autophagy regulates cellular homeostasis and cell viability and has protective roles against diseases; but in cancer, it plays the opposite role. The proteins involved in autophagy have been documented to exert a crucial role in cancer progression and are promising targets for TNBC therapy. However, it is still inconclusive how autophagy-related processes affect cancer development and progression. Our DEG analysis showed that expression levels of *ATG2B*, *ATG4A*, *ATG10*, *ATG14*, and *BECN1* decrease with an increase in the MP risk factor. Beclin-1 is frequently monoallelically deleted in breast cancer and interacts with ATG14 to trigger apoptosis inhibition in breast cancer, leading to cancer progression and chemotherapy resistance [24]. Overall survival analysis using KM plotter [25] indicated that low expression of these genes correlates with poor overall survival of breast cancer. On the other hand, with the increase in the MP risk factor, expression levels of *ATF2A* (activating transcription factor 2A), *ATG3*, *ATG4*, *ATG4B*, *ATG4C*, *ATG5*, *ATG9A*, *ATG9B*, *ATG12*, *ATG13*, and *ATG101* increased, suggesting their role in worsening breast cancer prognosis. Studies suggest that ATF4, ATG13, and ATG101 are involved in autophagosome initiation, and ATG5, ATG8, ATG12, and ATG16 family proteins are involved in expanding phagophore [24,26,27]. ATG5 plays an essential role and is often dysregulated in cancer cells [28]. Furthermore, higher *ATG3*, *ATG5*, and *ATG12* expression levels are associated with a lower overall survival rate in breast cancer patients, indicating a predictive value of these genes.

Similarly, the mercapturic acid pathway (MPy) is a subset of xenobiotic detoxification pathways (XDP) that are regulated by p53 (*TP53*) in response to xenobiotic stress. MPy consists of numerous GST isoenzymes and multiple phase III transporters of glutathione-electrophile conjugates (GS-E). Because phase III enzymes can catalyze the efflux of xenobiotics and their phase II metabolic end-products, they are critical determinants of the overall activity of MPy at both ends, regulating the concentration of the substrates of phase I and products of phase II enzymes. In cancer cells, GSH and the mercapturic acid pathway enzymes play a role in resistance to many chemotherapeutic drugs. MP High 2 subtype showed higher expression of *AKR1B1*, *CHAC1*, *CHOP*, *DPEP2*, *GCLC*, *GGT1*, *GLS*, *GOT1*, *GPX1*, *GSR*, *GSTA1*, *GSTA4*, *GSTP1*, *NFE2L2*, *KEAP1*, *RALBP1*, *SOD1*, *SOD2*, and *SOD3*. Overexpression of SOD1 and SOD3 in cancers helps to maintain cellular ROS under the critical threshold [29], and improve response to chemotherapy, respectively [30]. The DAVID analysis of these upregulated genes demonstrated the relationship between the GSH-linked stress defense and signaling pathways. Overexpression of these genes may contribute to increased detoxification of anticancer drugs. Cancer drug resistance is also related to the increased efflux of anticancer drugs due to the overexpression of the efflux transporters MRP1 and RALBP1. In High 2 or Basal groups, downregulation of *GSTA1*, *GSTM1*, *GSTM2*, and *GSTT1* gene expression can be correlated with the impaired ability to eliminate carcinogenic compounds and increased cancer risk. Furthermore, KM plotter survival analysis for *GSTM2* in breast cancer patients demonstrated a significantly poor overall survival associated with lower expression levels. Moreover, PC4 in PCA showed that *SOD2* and *GSTM2* were correlated in opposite directions and explained differences between Basal and High 2 groups as well as differences in other clinical factors. Nevertheless, several other studies suggest that it is unlikely that GSTs have a detrimental effect on breast cancer progression [31]. Therefore, the significant positive or negative influence of these genes on cancer progression may become evident when they are considered as part of a larger signaling cascade. This notion should be explored further in future studies.

GLYAT (Glycine N-acyltransferase) expression level was lower in MP High 2. In contrast, *MAP2K3* and *MAP3K7* expression was higher in MP High 2. GLYAT converts glycine to acyl glycine, and lower expression is correlated with poor overall survival of breast cancer patients [32]. MAP2K3 and MAP3K7 belong to the MAPK pathway, and activation of the MAPK pathway in breast cancer promotes proliferation, anti-apoptotic influence, and contributes to drug resistance, cancer cell survival, and invasion. Moreover, TUBB2B (tubulin beta 2B class IIb) is necessary for microtubule polymerization and depolymerization to spindle formation during mitosis. Its expression level was upregulated in MP High 2 but relatively unchanged in the BP Basal group. Since taxanes inhibit cell division by disrupting the mitotic spindle through the stabilization of microtubules, a high expression of TUBB2B could be a compensatory mechanism, however, this hypothesis needs to be addressed. Insulin-like growth factor-1 (IGF-1) is a growth factor and higher levels in the blood are linked to an increased risk of breast cancer and glioma-associated oncogene 1 (GLI1) is a critical transcriptional factor of the sonic hedgehog pathway that is linked with cancer progression and is related to the unfavorable prognosis of breast cancer. Breast cancer is linked with a high-fat diet and a sedentary lifestyle that leads to obesity, and increased estrogen levels and inflammation [33]. Both High 2 and Basal groups had increased expression of macrophage inflammation markers CD68 and CD163, also associated with obesity, which correlates with poor prognosis in breast cancer [34]. Our findings indicate a close interaction between these markers with other stress markers to increase risk factors for breast cancer.

Caspases belong to the cysteinyl aspartate-specific proteinase family and are associated with drug resistance, metastasis, apoptosis, and cell death. In our study, *CASP1*, *CASP2*, *CASP3*, *CASP4*, *CASP5*, *CASP7*, *CASP8*, *CASP9*, and *CASP10* expression levels are up in Basal and High 2 groups. It has been demonstrated previously that dysregulated caspase-3 is associated with breast cancer occurrence, invasion, and metastasis. In gastric cancer, caspase expression has prognostic significance including overall survival [35]. There is little or no evidence supporting a central role for individual caspases in cancer progression. Overexpression of these caspases in Basal and High 2 may have a combined apoptotic effect of all caspases rather than any single caspase in tumor progression.

Genetic alterations and epigenetic processes are major contributors to cancer development. Histone modification, regulated by histone deacetylases (HDAC), plays a crucial role in the epigenetic regulation of gene expression involved in breast cancer growth, proliferation, and metastasis. Recently, several novel HDAC inhibitors have been developed to inhibit breast cancer cell proliferation, which influences mitosis and DNA repair. HDAC11 expression level is significantly low in the High 2 and Basal groups, which can be correlated with the poor overall survival of patients. Expression levels of *HDAC1*, *HDAC2*, *HDAC3*, *HDAC5*, *HDAC6*, *HDAC8*, *HDAC9*, and *HDAC11* are relatively higher in the High 2 and Basal groups. HDAC2 is associated with poor survival and relapse of cancer, HDAC3 promotes oncogenesis and is a good target for therapeutics, HDAC5 is known to increase the stemness of breast cancer cells, and HDAC7 protein is associated with reoccurrence of breast cancer.

Our comprehensive analysis provides novel insights into gene networks and pathways associated with breast cancer and their association with clinical parameters; however, there are several limitations to consider. First, while the study included 978 patients, certain subgroups (e.g., Hispanic) were not represented, which limits the ability to draw any conclusions for this ethnicity. Second, the study focused on a predefined set of 111 genes related to specific pathways. A more comprehensive whole transcriptome analysis may reveal additional important genes/pathways. Third, as a retrospective analysis of existing data, there are inherent limitations in terms of potential selection bias and inability to control all confounding variables. Fourth, the control group on this study was represented by lowest-risk cancer cells as opposed to normal cells because the FLEX registry is designed to consent cancer patients for participation and provides no control group with normal tissue. This phenomenon can potentially mask some relationships between these genes that may be differentially expressed from benign or normal tissue but may not be picked up between two types of cancer tissue. Finally, while the study identified differentially expressed genes corrected with functional outcomes, there was no functional validation of the roles of these genes in breast cancer progression or treatment response. This warrants future exploration.

## 5. Conclusions

In conclusion, this work highlights the differences in the selected gene expression levels in high-risk groups of patients and molecular subtypes. Our analysis revealed that autophagy, apoptosis, histone deacetylation, and oxidative stress-related gene expression levels were mainly increased with the worsening of the cancer. We identified eight to nine DEGs that could be used as possible biomarkers of risk determinants regardless of the molecular subtype. Protein–protein interaction analysis of these gene products provides us a framework to recognize previously underappreciated oncogenic drivers. Furthermore, aberrant epigenetic, oxidative stress, inflammatory, and autophagic alterations play a decisive role in cancer initiation and propagation via the regulation of key tumor suppressor genes and oncogenes or by modulation of essential signaling pathways. A chronically altered gene expression, even if moderate compared with the effects of a highly modified gene expression, is expected to cause a sustained augmentation of multiple downstream defense and repair mechanisms and thus to have a cumulative effect on carcinogenic susceptibility and lineage plasticity. Therefore, a clinically relevant corollary of this study is that identifying such markers can serve as a prognostic and predictive biomarker in breast cancer. Furthermore, our study also establishes a strong correlation between age, BMI, race, menopausal status, intrinsic molecular subtypes, and risk of recurrence. This underscores the importance of integrating clinical characteristics with molecular profiling to improve personalized treatment strategies and outcomes for breast cancer patients.

## Figures and Tables

**Table 1:** Baseline characteristics.

Variable	Mean ± SD [*n* (%)]
**Age**	58.84 ± 13.04
**BMI**	30.04 ± 7.34
**Race**	
White	799 (81.7)
Black	123 (12.6)
Other *	56 (5.7)
**Menopausal status**	
Post	717 (73.3)
Peri	128 (13.1)
Pre	133 (13.6)
**ER status**	
Negative	265 (27.1)
Positive	713 (72.9)
**PR status**	
Negative	265 (27.1)
Positive	590 (60.5)
**HER2 status**	
Negative	721 (73.7)
Positive	257 (26.3)
**Risk of recurrence**	
Ultralow	76 (7.8)
Low	176 (18)
High 1	315 (32.2)
High 2	411 (42)
**Intrinsic molecular subtypes**	
Luminal A	250 (25.6)
Luminal B	250 (25.6)
HER2	228 (23.3)
Basal	250 (25.6)

* Includes Asian, Native American, Hispanic, Hawaiian, or Pacific Islander.

**Table 2:** Functional annotation of input genes.

GO	Description	Count	%	−Log10(P)	−Log10(q)
GO:0000422	autophagy of mitochondrion	18	16.22	29.56	25.39
GO:0006749	glutathione metabolic process	17	15.32	28.03	24.15
GO:0062197	cellular response to chemical stress	24	21.62	25.58	22.19
GO:0019752	carboxylic acid metabolic process	30	27.03	21.57	18.47
GO:0043525	positive regulation of neuron apoptotic process	14	12.61	20.36	17.35
GO:0009410	response to xenobiotic stimulus	23	20.72	19.73	16.82
GO:0031667	response to nutrient levels	23	20.72	18.64	15.82
GO:1901699	cellular response to nitrogen compound	23	20.72	16.02	13.33
GO:0071216	cellular response to biotic stimulus	15	13.51	13.68	11.12
GO:0071214	cellular response to abiotic stimulus	15	13.51	12.09	9.69
GO:0097193	intrinsic apoptotic signaling pathway	12	10.81	12.04	9.66
GO:0006476	protein deacetylation	6	5.41	10.99	8.64
GO:2001233	regulation of apoptotic signaling pathway	15	13.51	10.73	8.4
GO:0050727	regulation of inflammatory response	15	13.51	10.3	8.01
GO:0034976	response to endoplasmic reticulum stress	12	10.81	10.29	8.01
GO:0010506	regulation of autophagy	14	12.61	10.24	7.97
GO:0030162	regulation of proteolysis	17	15.32	10.14	7.88
GO:0070848	response to growth factor	16	14.41	10.08	7.84
GO:2000377	regulation of reactive oxygen species metabolic process	10	9.01	9.69	7.47
GO:0048511	rhythmic process	12	10.81	9.49	7.28

The table includes significantly enriched (adjusted *p*-value < 0.05) GO biological processes among the 111 input genes using Metascape, https://metascape.org/.

## Data Availability

All data are included in the article.

## Supplementary Materials

The following supporting information can be downloaded at: https://www.mdpi.com/article/10.3390/biomedicines13051058/s1, Table S1: MammaPrint and BluePrint category distribution of the FLEX samples used in this study; Table S2: Summary of one-way ANOVA for principal components and clinical variables; Table S3: Summary of linear models for principal components and clinical variables; Table S4: Gene Expression Levels for MammaPrint and BluePrint Subtypes. (See attached Excel File); Table S5: High2 vs Ultralow: KEGG enrichment analysis; Table S6: Basal vs Luminal A: KEGG enrichment analysis; Figure S1: Relationship between Principal Component (PC) scores and baseline variables; Figure S2: The overall survival significance in breast cancer (www.kmplot.com) of top and bottom 3 gene expression differences considering MP High2 and Ultralow comparison; Figure S3: The overall survival significance in breast cancer (www.kmplot.com) of top and bottom 3 gene expression differences considering BP Basal and Luminal A comparison; Figure S4: The overall survival significance of autophagy-related gene expression in breast cancer (www.kmplot.com); Figure S5: Pathway enrichment among significantly different genes; Figure S6: Interactome analysis for upregulated genes; Figure S7: Interactome analysis for downregulated genes.

## Abbreviations

DCIS: Ductal carcinoma in situ; ER: Estrogen receptor; PR: Progesterone receptor; TNBC: Triple Negative Breast Cancer; NAC: Neoadjuvant chemotherapy; ACT: Adjuvant chemotherapy; MP: MammaPrint^®^; BP: BluePrint^®^; PCA: Principal component analysis; KEGG: Kyoto Encyclopedia Gene and Genome; ROS: Reactive oxygen species; DEG: Differential gene expression; OS: Overall survival; ER: Estrogen receptor; HER2: Epidermal growth factor receptor 2.
