# Supplementary material for: Relevance of Cellular Homeostasis-Related Gene Expression Signatures in Distinct Molecular Subtypes of Breast Cancer
Source: Biomedicines. 2025 Apr 28;13(5):1058. doi: 10.3390/biomedicines13051058 (PMC12108624; doi:10.3390/biomedicines13051058)
Supplement: Supplementary file 1 [file biomedicines-13-01058-s001.zip › biomedicines-3566910-supplementary.pdf]

Supplemental Material

Table S1. MammaPrint and BluePrint category distribution of the FLEX samples used in this study.

	Luminal A % (n)	Luminal B % (n)	HER2 % (n)	Basal % (n)	Total (n)
**Ultralow**	30.40% (76)	0.00% (0)	0.00% (0)	0.00% (0)	76
**Low**	69.60% (174)	0.00% (0)	0.88% (2)	0.00% (0)	176
**High 1**	0.00% (0)	87.20% (218)	36.40% (83)	5.60% (14)	315
**High 2**	0.00% (0)	12.80% (32)	62.72% (143)	94.40% (236)	411
**Total n**	250	250	250	228	**978**

978 FLEX samples representing ~25% of each genomic subtype according to MammaPrint. FLEX (NCT03053193) study includes stage I-III breast cancer patients who receive MammaPrint (with or without BluePrint) as the standard of care and consent to whole transcriptome and clinical data collection. Transcriptome is based on microarray probe intensities. MammaPrint is a 70-gene risk of distant metastasis signature that classifies patients into risk categories: Ultralow, Low, High 1, High 2. BluePrint is an 80-gene molecular subtyping signature, which categorizes tumors as Luminal-, HER2-, Basal-Type.

Table S2. Summary of one-way ANOVA for principal components and clinical variables.

Principal component	Variable	Sum of Squares	Mean Square	F value	p-value
PC 1	Race	389	194.44	5.03 $_{(2, 975)}$	**0.007**
	Menopausal Status	289	144.27	3.723 $_{(2, 975)}$	**0.025**
	Intrinsic molecular subtypes	213	70.85	1.822 $_{(3, 974)}$	0.141
	Risk of recurrence	225	74.91	1.928 $_{(3, 974)}$	0.123
PC 2	Race	34	17.1	1.603 $_{(2, 975)}$	0.202
	Menopausal Status	10	4.908	0.459 $_{(2, 975)}$	0.632
	Intrinsic molecular subtypes	114	37.96	3.582 $_{(3, 974)}$	**0.014**
	Risk of recurrence	405	135.1	13.11 $_{(3, 974)}$	**<0.001**
PC 3	Race	17	8.526	1.576 $_{(2, 975)}$	0.202
	Menopausal Status	27	13.5	2.501 $_{(2, 975)}$	0.632
	Intrinsic molecular subtypes	307	102.47	20.02 $_{(3, 974)}$	**0.014**
	Risk of recurrence	303	101.16	19.75 $_{(3, 974)}$	**<0.001**
PC 4	Race	75	37.45	8.364 $_{(2, 975)}$	**<0.001**
	Menopausal Status	107	53.56	12.05 $_{(2, 975)}$	**<0.001**
	Intrinsic molecular subtypes	2947	982.3	640.7 $_{(3, 974)}$	**<0.001**
	Risk of recurrence	2485	828.4	412.7 $_{(3, 974)}$	**<0.001**
PC 5	Race	11.1	5.553	1.942 $_{(2, 975)}$	0.144
	Menopausal Status	34	17.021	6.001 $_{(2, 975)}$	**0.002**
	Intrinsic molecular subtypes	512.3	170.78	72.72 $_{(3, 974)}$	**<0.001**
	Risk of recurrence	185.4	61.81	23.03 $_{(3, 974)}$	**<0.001**

F value-f statistics and degrees of freedom in parentheses

Table S3. Summary of linear models for principal components and clinical variables.

Principal component	Variable	β	Standard Error	p-value
PC 1	Age	0.038	0.015	**0.012**
	BMI	0.028	0.027	0.307
PC 2	Age	-0.001	0.008	0.921
	BMI	-0.007	0.014	0.611
PC 3	Age	-0.001	0.006	0.844

	BMI	-0.002	0.010	0.873
PC 4	Age	-0.030	0.005	**<0.001**
	BMI	0.031	0.009	**0.001**
PC 5	Age	-0.017	0.004	**<0.001**
	BMI	0.000	0.007	0.955

BMI-body mass index

Table S4. Gene Expression Levels for MammaPrint and BluePrint Subtypes. (See attached Excel File)

Table S5. High2 vs Ultralow: KEGG enrichment analysis.

Enrichment FDR	nGenes	Pathway Genes	Fold Enrichment	Pathway	URL	Genes
6.213E-05	3	57	133.8	Path:hsa00480 Glutathione metabolism	http://www.genome.jp/kegg-bin/show_pathway?hsa00480	GSTM1 GSTM2 CHAC1
3.039E-03	2	68	74.8	Path:hsa05204 Chemical carcinogenesis-DNA adducts	http://www.genome.jp/kegg-bin/show_pathway?hsa05204	GSTM1 GSTM2
3.039E-03	2	69	73.7	Path:hsa00982 Drug metabolism-cytochrome P450	http://www.genome.jp/kegg-bin/show_pathway?hsa00982	GSTM1 GSTM2
3.039E-03	2	73	69.7	Path:hsa01524 Platinum drug resistance	http://www.genome.jp/kegg-bin/show_pathway?hsa01524	GSTM1 GSTM2
3.039E-03	2	75	67.8	Path:hsa00980 Metabolism of xenobiotics by cytochrome P450	http://www.genome.jp/kegg-bin/show_pathway?hsa00980	GSTM1 GSTM2
3.039E-03	2	79	64.4	Path:hsa00983 Drug metabolism-other enzymes	http://www.genome.jp/kegg-bin/show_pathway?hsa00983	GSTM1 GSTM2
7.165E-03	2	138	36.8	Path:hsa05418 Fluid shear stress and atherosclerosis	http://www.genome.jp/kegg-bin/show_pathway?hsa05418	GSTM1 GSTM2
1.874E-03	3	223	34.2	Path:hsa05208 Chemical carcinogenesis-reactive oxygen species	http://www.genome.jp/kegg-bin/show_pathway?hsa05208	GSTM1 GSTM2 SOD2
9.031E-03	2	167	30.4	Path:hsa05225 Hepatocellular carcinoma	http://www.genome.jp/kegg-bin/show_pathway?hsa05225	GSTM1 GSTM2
1.084E-02	2	197	25.8	Path:hsa05207 Chemical carcinogenesis-receptor activation	http://www.genome.jp/kegg-bin/show_pathway?hsa05207	GSTM1 GSTM2
1.178E-02	2	214	23.8	Path:hsa05417 Lipid and atherosclerosis	http://www.genome.jp/kegg-bin/show_pathway?hsa05417	CXCL8 SOD2
5.963E-03	3	530	14.4	Path:hsa05200 Pathways in cancer	http://www.genome.jp/kegg-bin/show_pathway?hsa05200	GSTM1 GSTM2 CXCL8
9.031E-03	4	1538	6.6	Path:hsa01100 Metabolic pathways	http://www.genome.jp/kegg-bin/show_pathway?hsa01100	CBS GSTM1 GSTM2 CHAC1

Table S6. Basal vs Luminal A: KEGG enrichment analysis.

Enrichment FDR	nGenes	Pathway Genes	Fold Enrichment	Pathway	URL	Genes
6.456E-05	3	57	133.8	Path:hsa00480 Glutathione metabolism	http://www.genome.jp/kegg-bin/show_pathway?hsa00480	GSTM1 GSTM2 CHAC1
2.763E-03	2	68	74.8	Path:hsa05204 Chemical carcinogenesis-DNA adducts	http://www.genome.jp/kegg-bin/show_pathway?hsa05204	GSTM1 GSTM2
2.763E-03	2	69	73.7	Path:hsa00982 Drug metabolism-cytochrome P450	http://www.genome.jp/kegg-bin/show_pathway?hsa00982	GSTM1 GSTM2
2.763E-03	2	73	69.7	Path:hsa01524 Platinum drug resistance	http://www.genome.jp/kegg-bin/show_pathway?hsa01524	GSTM1 GSTM2
2.763E-03	2	75	67.8	Path:hsa00980 Metabolism of xenobiotics by cytochrome P450	http://www.genome.jp/kegg-bin/show_pathway?hsa00980	GSTM1 GSTM2
2.763E-03	2	79	64.4	Path:hsa00983 Drug metabolism-other enzymes	http://www.genome.jp/kegg-bin/show_pathway?hsa00983	GSTM1 GSTM2
7.446E-03	2	138	36.8	Path:hsa05418 Fluid shear stress and atherosclerosis	http://www.genome.jp/kegg-bin/show_pathway?hsa05418	GSTM1 GSTM2
1.298E-03	3	223	34.2	Path:hsa05208 Chemical carcinogenesis-reactive oxygen species	http://www.genome.jp/kegg-bin/show_pathway?hsa05208	GSTM1 GSTM2 SOD2
9.385E-03	2	167	30.4	Path:hsa05225 Hepatocellular carcinoma	http://www.genome.jp/kegg-bin/show_pathway?hsa05225	GSTM1 GSTM2
1.127E-02	2	197	25.8	Path:hsa05207 Chemical carcinogenesis-receptor activation	http://www.genome.jp/kegg-bin/show_pathway?hsa05207	GSTM1 GSTM2
1.224E-02	2	214	23.8	Path:hsa05417 Lipid and atherosclerosis	http://www.genome.jp/kegg-bin/show_pathway?hsa05417	CXCL8 SOD2
8.665E-04	4	530	19.2	Path:hsa05200 Pathways in cancer	http://www.genome.jp/kegg-bin/show_pathway?hsa05200	GLI1 GSTM1 GSTM2 CXCL8
9.385E-03	4	1538	6.6	Path:hsa01100 Metabolic pathways	http://www.genome.jp/kegg-bin/show_pathway?hsa01100	CBS GSTM1 GSTM2 CHAC1

Figure S1. Relationship between Principal Component (PC) scores and baseline variables. Variables considered for group comparisons were A. age, B. BMI, C. race, D. menopausal status, E. BluePrint-80-gene expression based intrinsic molecular subtypes, and F. MammaPrint, the 70-gene signature, categories of risk of recurrence. Box plots annotated with an asterisk indicate statistical significance after adjusting for multiple comparisons using the Holm-Bonferroni approach (* <0.05, **<0.01, ***<0.001). The significance level was set at p = 0.05.

Supplemental Figure 1A.

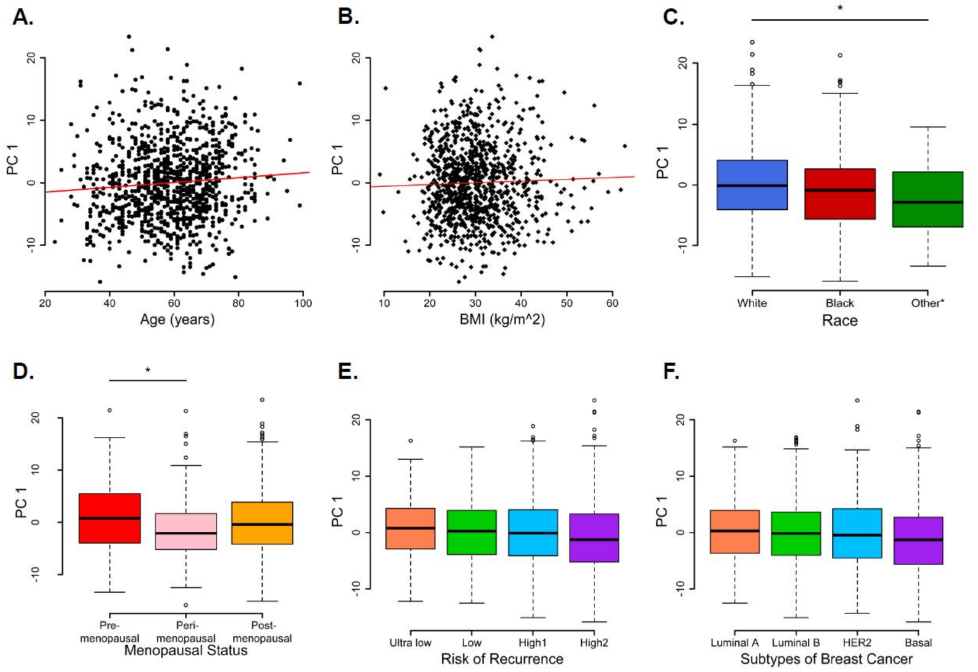

A) Relationship between Principal Component 1 Scores and Baseline Variables.

Supplemental Figure 1B.

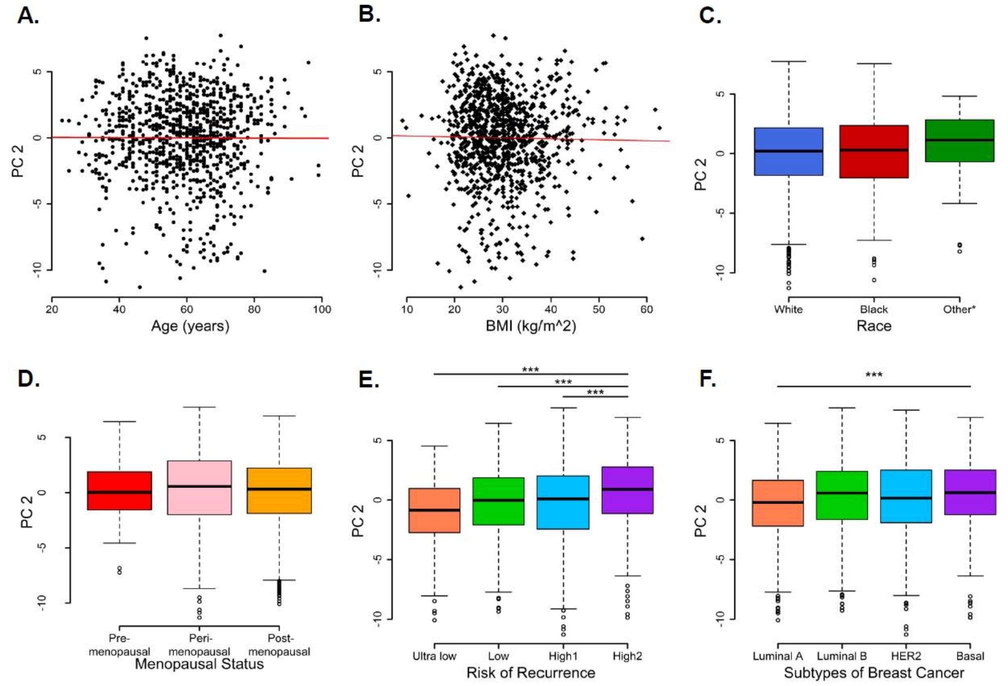

B) Relationship between Principal Component 2 Scores and Baseline Variables.

Supplemental Figure 1C.

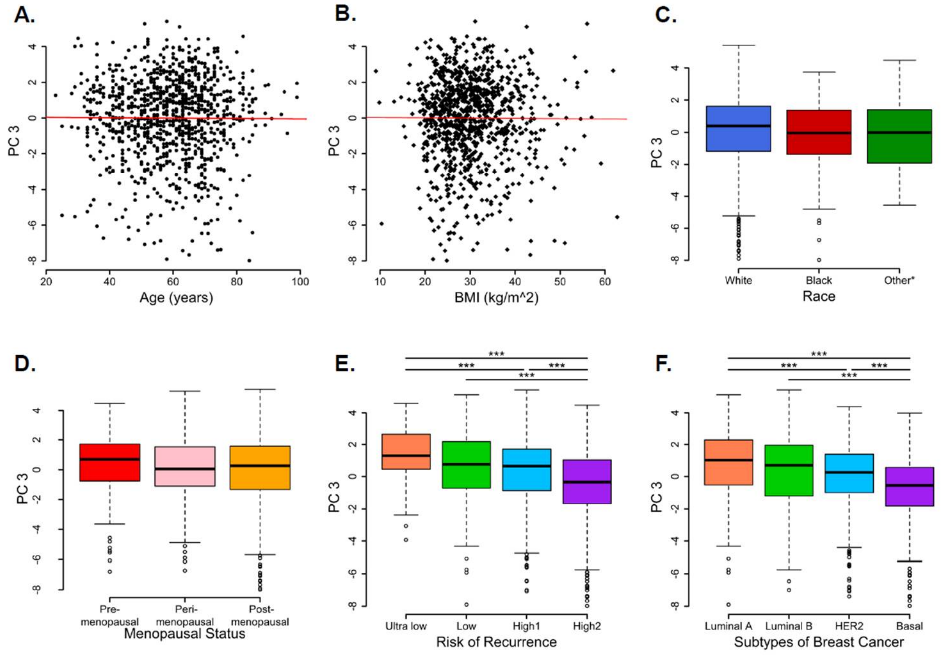

C) Relationship between Principal Component 3 Scores and Baseline Variables.

Supplemental Figure 1D.

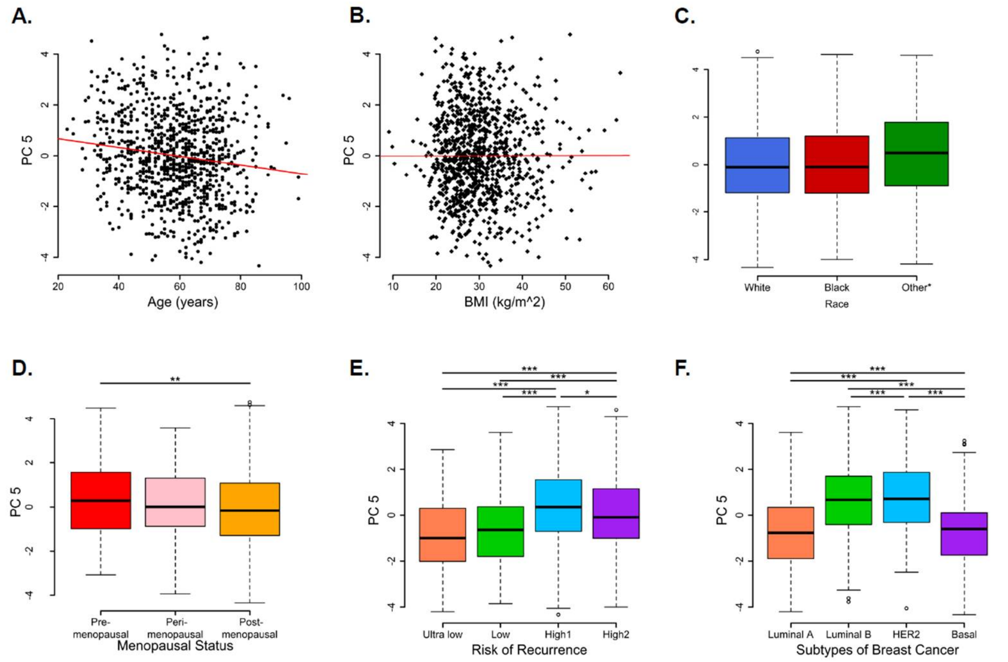

D) Relationship between Principal Component 5 Scores and Baseline Variables.

Supplemental Figure 2.

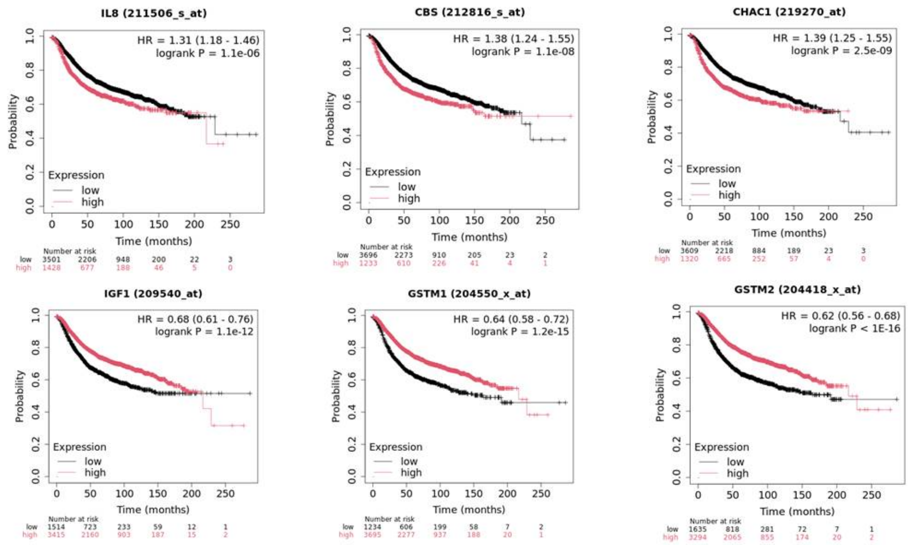

Figure S2. The overall survival significance in breast cancer (www.kmplot.com) of top and bottom 3 gene expression differences considering MP High2 and Ultralow comparison.

Supplemental Figure 3.

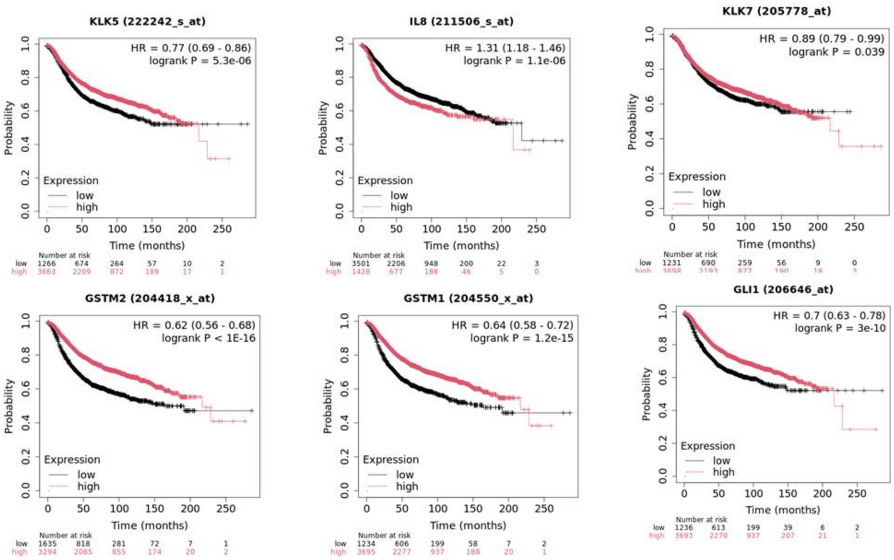

Figure S3. The overall survival significance in breast cancer (www.kmplot.com) of top and bottom 3 gene expression differences considering BP Basal and Luminal A comparison.

Supplemental Figure 4.

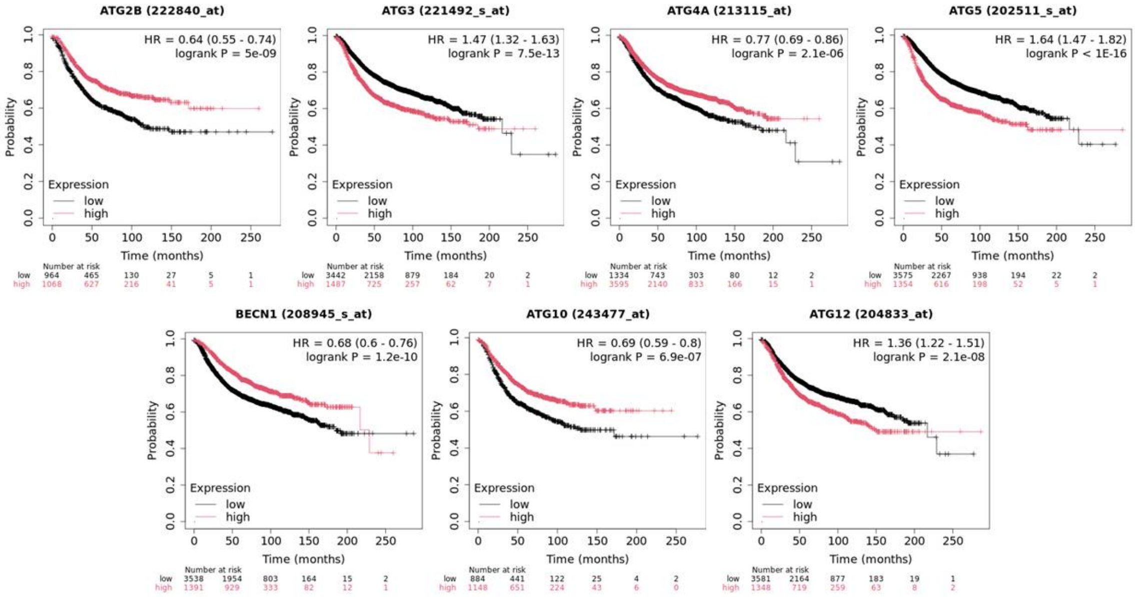

Figure S4. The overall survival significance of autophagy-related gene expression in breast cancer (www.kmplot.com).

Supplemental Figure 5.

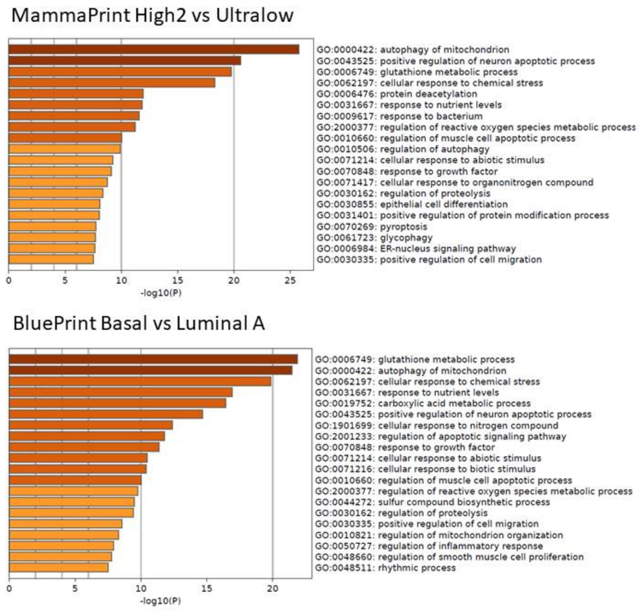

Figure S5. Pathway enrichment among significantly different genes. Input genes are significantly different genes from MammaPrint High2 vs Ultralow comparison (n=78) for the above plot and BluePrint Basal vs Luminal A comparison (n=78) for the below plot. 67 genes are similar between the two input gene lists.

Supplemental Figure 6.

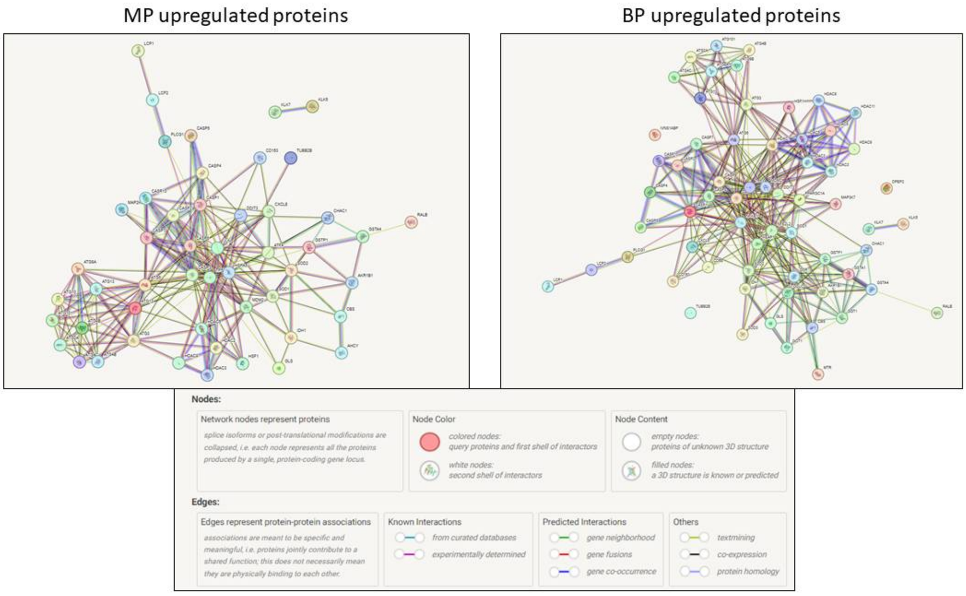

Figure S6. Interactome analysis for upregulated genes. A total of 67 upregulated BP and 51 MP differentially expressed genes (DEGs) with a p-adj < .05 were analyzed in the STRING tool. Each node (circle) represents the protein encoded by the gene.

Supplemental Figure 7.

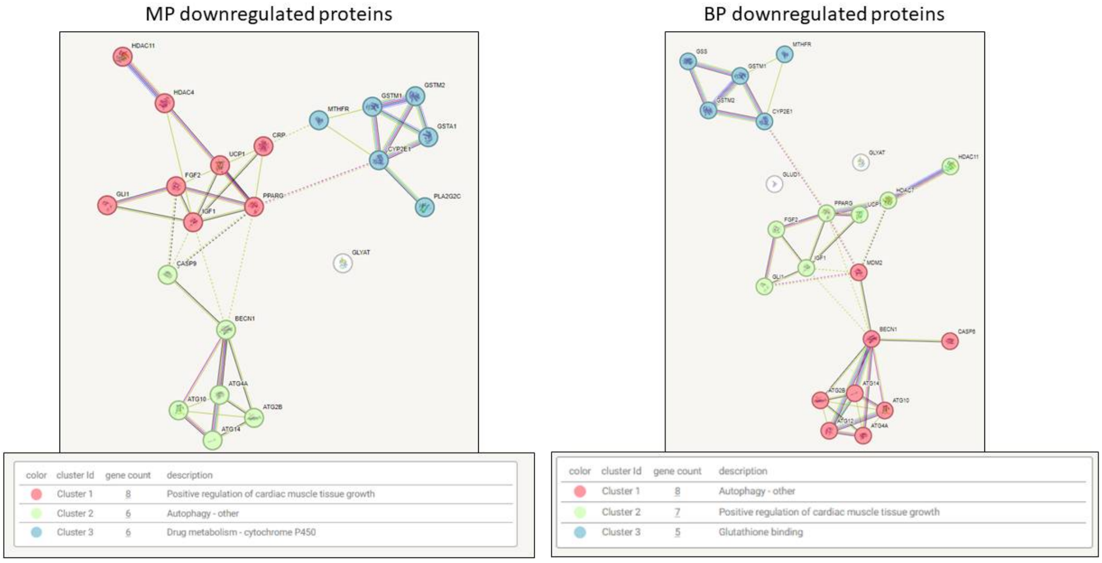

Figure S7. Interactome analysis for downregulated genes. A total of 25 downregulated BP and 23 MP differentially expressed genes (DEGs) with a p-adj < .05 were analyzed in the STRING tool. Each node (circle) represents the protein encoded by the gene.
